# Case Report: A novel *WRN* mutation in Werner syndrome patient with diabetic foot disease and myelodysplastic syndrome

**DOI:** 10.3389/fendo.2022.918979

**Published:** 2022-07-15

**Authors:** Huifang Peng, Jie Wang, Yanyun Liu, Haiping Yang, Liping Li, Yujin Ma, Huiqin Zhuo, Hongwei Jiang

**Affiliations:** ^1^ Endocrine and Metabolic Disease Center, Medical Key Laboratory of Hereditary Rare Diseases of Henan, Luoyang Sub-Center of National Clinical Research Center for Metabolic Diseases, The First Affiliated Hospital, and College of Clinical Medicine of Henan University of Science and Technology, Luoyang, China; ^2^ Department of Hematology, The First Affiliated Hospital, and College of Clinical Medicine of Henan University of Science and Technology, Luoyang, China; ^3^ Department of Gastrointestinal Surgery, Zhongshan Hospital of Xiamen University, Xiamen, China

**Keywords:** Werner syndrome, myelodysplastic syndrome (MDS), diabetic foot disease, *WRN* gene, novel mutation

## Abstract

Werner syndrome is an autosomal recessive rare disease caused by a *WRN* gene mutation, which is rarely reported in the Chinese population. We report the clinical and genetic data of a Chinese patient with Werner syndrome. The proband was a 40-year-old male patient who presented with diabetic foot ulcers, accompanied by short stature, cataracts, hypogonadism, and hair thinning, and myelodysplastic syndrome (MDS) occurred after 18 months. Genetic sequencing showed there were compound heterozygous mutations as c.3384-1G>C and c.3744dupA in the *WRN* gene. The c.3744dupA mutation is a novel pathogenic variation for Werner syndrome.

## Introduction

WRN, belonging to the DNA helicases family, is associated with DNA glycosylase, nonhomologous end joining (NEIL1), base excision repair (BER), and homologous recombination (HR) (1). Werner syndrome (the Online Mendelian Inheritance in Man #277700) was caused by *WRN* gene mutation as autosomal recessive in humans, with mainly clinical symptoms as type 2 diabetes mellitus, hypogonadism, osteoporosis, atherosclerosis and malignancies, short stature, and other common age-related diseases (2), and the reports in a Chinese population were just a few (3). In this study, we report a 40-year-old man who was hospitalized due to diabetic foot ulcers, with short stature, sparse hair, and uneven fat distribution, and with a history of cataracts, osteonecrosis of the femoral head, and supraventricular tachycardia and hypophysis. Genetic test results showed that there were compound heterozygous mutations of *WRN* c.3384-1G>C and c.3744dupA (p.Ala1248fs) in the proband. The proband was diagnosed with Werner syndrome, while during follow-up, 18 months later, the patient developed myelodysplastic syndrome (MDS) and was hospitalized again.

## Case

**Figure 1:**
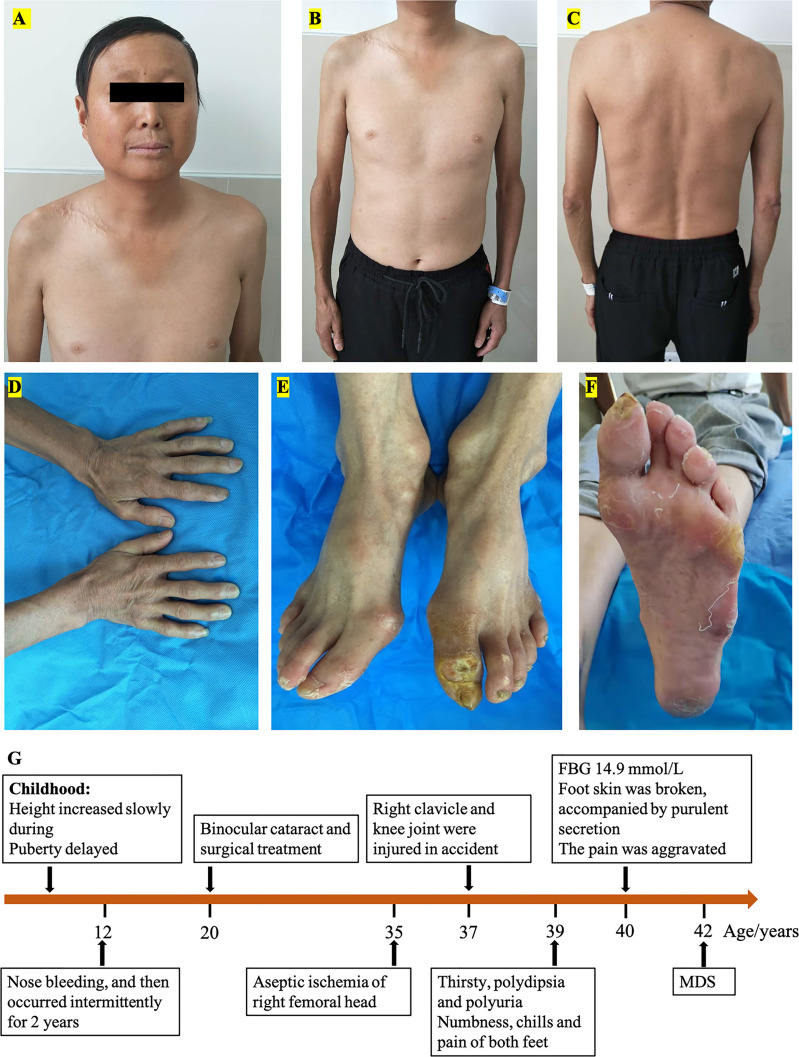
Patient’s characteristics: **(A)** face, **(B)** ventral trunk, **(C)** back of the trunk, **(D)** hands, **(E)** feet, **(F)** sole, and clinical history diagram **(G)**.

The proband, a 40-year-old man, was hospitalized with a diabetic foot ulcer as the main complaint. Physical examination showed that he was 147 cm tall, weighed 38 kg, had a body mass index (BMI) of 17.6 kg/m^2^, a temperature of 36.7°C, a respiratory rate of 16 breaths/min, a blood pressure of 121/82 mmHg, a pulse rate of 97 beats/min, and had lost 5 kg in the previous 3 months. His hair, eyebrows, and beard were sparse (**Figure 1A**). The limbs were slender and out of proportion to the trunk, and with reduced subcutaneous fat (**Figures 1B, C**). The skin of the face, hands, and feet was waxy, thin, and with low elasticity (**Figures 1D, E**). The stretched penile length was 6 cm, the bilateral testicles were 2 ml, and the pubic hair Tanner stage was 1–2. There were 0.8 cm × 0.8 cm wounds with purulent secretion on the dorsal side of the left toe and 0.5 cm × 0.5 cm wounds at the lateral metatarsal joint of the left foot (**Figures 1E, F**). There was no deformity of the chest and spine. Clinical history (**Figure 1G**) was as follows: the height of the patient increased more slowly than their peers since childhood and delayed puberty. Nose bleeding was caused by trauma at age of 12 and then occurred intermittently for 2 years. Binocular cataracts occurred at the age of 20 with surgical treatment. Aseptic ischemia of the right femoral head was detected at the age of 35. At the age of 37, the right clavicle and knee joint were injured in a car accident and underwent surgical treatment. Thirsty, polydipsia, and polyuria occurred at the age of 39 and were not treated. Furthermore, consequential symptoms such as numbness, chills, and pain in both feet were not addressed and thus went untreated. Three months before this time of hospitalization, he received a test of fasting blood glucose (FBG) at 14.9 mmol/L (reference values 3.90–6.10 mmol/L) in the outpatient department, and metformin and Xiaoke pills were taken. Two months before this time of hospitalization, the foot skin was broken, accompanied by purulent secretion, and the pain was aggravated, so he went to the Endocrinology Department of our hospital. Family history has shown that there was no similar patient, and both parents and two older sisters were healthy.

### Laboratory tests

There were some abnormal indexes of glucose metabolism and islet function, serum lipids, thyroid function, adrenal function, liver and kidney function, and sexual hormones, and the results are shown in **Table 1**. Diabetes-related antibodies were negative, including insulin autoantibody, islet cell antibody, and glutamic acid decarboxylase autoantibody. The culture of secretion bacteria from feet wounds showed Shigella. Muck’s routine examination demonstrated occult blood. Electrocardiogram results showed sinus rhythm with 88 beats/min and ventricular preshock type A. Peripheral nerve damage of the lower limbs was detected using an electromyogram, which showed the following (1): the sensory conduction velocity of the right sural nerve decreased (ankle-lower leg, 170.00 mm, 39.00 m/s) and the latency prolonged (4.40 ms); (2) the velocities of bilateral median and ulnar nerves were at the lower limit of normal value; (3) the amplitude of motor conduction of the left median (wrist-elbow, 190.00 mm, elbow 5.81 mV) and ulnar nerves (wrist-elbow, 220.00 mm, elbow 5.62 mV) decreased, and distal latency prolonged (median elbow 9.20 ms, ulnar elbow 8.20 ms); (4) the motor conduction velocity (36 m/s) and amplitude (ankle 0.22 mV) of the right peroneal nerve decreased, and the distal latency prolonged (fibula-head 13.30 ms); (5) left peroneal and tibial nerves motor conduction potential wave were not extracted. The results of color Doppler ultrasound showed that the patient had fatty liver, intrahepatic bile duct stones, testicular volume reduction with testicular microlithiasis, and no obvious abnormality was found in the heart and lower limb arteries.

**Table 1:** Laboratory investigations of the proband.

Items	Results	Reference values
Blood glucose (mmol/L)		
Fasting	7.13	3.90–6.10
30 min after OGTT	12.39	3.90–11.10
60 min after OGTT	17.75	6.70–9.40
120 min after OGTT	18.33	3.90–7.80
180 min after OGTT	18.04	3.90–6.70
C-Peptide (ng/ml)		
Fasting	2.21	1.10–4.40
30 min after OGTT	4.47	–
60 min after OGTT	7.23	–
120 min after OGTT	12.69	–
180 min after OGTT	11.98	–
HbA1c (%)	8.00	4.50–6.50
Urine glucose	+++	–
Serum lipids		
Triacylglycerol (mmol/L)	11.19	0.90–1.72
Total cholesterol (mmol/L)	9.06	3.40–5.17
LDL-c (mmol/L)	3.05	2.59–3.34
HDL-c (mmol/L)	2.23	1.16–1.42
Thyroid function test		
TSH (μIU/ml)	7.49	0.55–4.78
FT3 (pg/ml)	2.46	2.30–4.20
FT4 (ng/dl)	0.96	0.89–1.76
TPOAb (IU/ml)	<28.00	–
Adrenal function		
ACTH (pg/ml)	44.60	–
Cor (8 am) (μg/dl)	39.00	4.80–20.60
Cor (16 am) (μg/dl)	12.90	4.80–20.60
Cor (0 am) (μg/dl)	3.30	4.80–20.60
Serum sexual hormones		
FSH (mIU/ml)	30.40	1.70–7.70
LH (mIU/ml)	8.17	2.10–14.70
PRL (ng/ml)	8.13	1.90–25.00
T (ng/dl)	53.54	262.00–1,593.00
E2 (pg/ml)	12.15	(0.00–56.00)
P (ng/ml)	0.47	(0.28–1.22)
HCG excitation test (T (ng/dl))		
−15 min	40.62	–
0 min	41.56	–
24 h	50.26	–
48 h	62.12	–
72 h	66.67	–
Blood routine examination		
Hb	117.00	110.00–150.00
Platelet (×10^9^/L)	352.00	125.00–350.00
Serum GGT (U/L)	77.00	10.00–60.00
Serum creatinine (μmol/L)	78.91	59.00–104.00
Urine protein	+	–

+, positive; +++, strongly positive.

**Figure 2:**
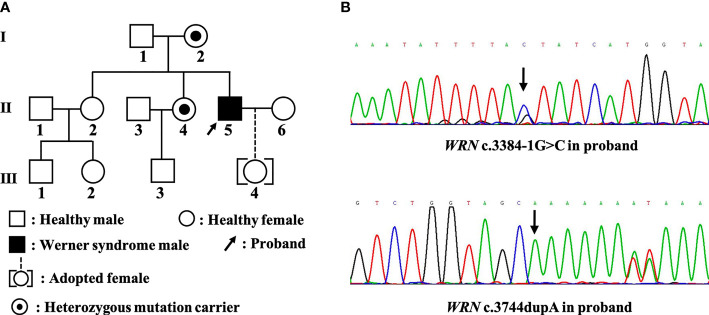
**(A)** Family map and **(B)** Sanger sequencing results.

The karyotype result for the first time showed a completely normal male karyotype as 46,XY from peripheral blood cells. The whole exome sequencing (WES) for proband (**Figure 2A** **, Ⅱ-5**) using peripheral blood demonstrated that there are heterozygous variants of the *WRN* gene (NM_000553) c.3384-1G>C (GRCh37, Chr8:31004568) and c.3744dupA p.Ala1248fs (**Figure 2B**). Family Sanger sequencing showed that the mother and younger sister (**Figures 2A** **, Ⅰ-2, Ⅱ-4**) had the same c.3384-1G>C variant. The c.3744dupA variants were not detected in the mother and two sisters. The father (**Figure 2A** **, Ⅰ-1**) refused any genetic testing. Although we do not know the genetic status of the patient’s father, the c.3384-1G>C variant has been detected in two family members (mother and younger sister), so we believe that the patient carries compound heterozygous mutations of c.3384-1G>C and c.3744dupA in the *WRN* gene. The c.3384-1G>C was a splice mutation, and according to the American College of Medical Genetics and Genomics/Association for Molecular Pathology (ACMG/AMP) variant pathogenicity guidelines (4), this mutation was judged to be pathogenic (PVS1+PM2+PP3). The c.3744dupA mutation, which was not included in the gnomAD database and the frequency in people was not known, was judged to be pathogenic (PVS1+PM2+PM3).

### Diagnosis, treatments, and follow-up

Combined with clinical manifestations and WES results, the patient was diagnosed with Werner syndrome caused by *WRN* gene mutations. For glucose control, insulin was used at 45 IU in total daily, metformin tablets at 0.5 g three times a day, pioglitazone dispersible tablets at 15 mg twice a day before breakfast and dinner, mecobalamin at 0.5 mg three times daily, and aspirin enteric-coated tablets at 0.1 g every night. Debridement and anti-infection treatments were carried out for diabetic foot disease, and the patient left the hospital after 40 days of treatments.

Eighteen months later, he was hospitalized in the Hematology Department, and the MDS was definitely diagnosed by bone marrow puncture morphology, biopsy, and chromosome karyotype analysis. The second time of karyotype analysis showed that the chromosomes had structural and numerical abnormalities in marrow cells as 45~46,XY,add(3)(p13),−5,del(7)(q22),+8,−20,+marl,+mar2[cp12]/46,XY[1]. The patient refused chemotherapy and instead received allogeneic red blood cells and erythropoietin to treat anemia, cefuroxime to treat infection, and an ibuprofen capsule as an analgesic. Patients have been informed of the severe adverse prognosis of Werner syndrome and need to pay attention to how their condition changes over time.

## Discussion

The WRN protein contains an N-terminal 3′ to 5′ exonuclease domain, an ATP-dependent helicase domain, and a RecQ helicase domain in the central region and a helicase RNase D C-terminal domain and a nuclear localization signal (5). The c.3744dupA mutation, detected in this patient, might affect the nuclear localization signal of WRN helicase since it is a frameshift mutation. Also, this mutation was not reported before and is not contained in the gnomAD database. We wanted to say that WRN c.3744dupA was a novel pathogenic mutation for Werner syndrome.

An extremely high prevalence of diabetes mellitus was mentioned in Werner syndrome. Over half of the patients with the clinical features of diabetes mellitus or impaired glucose tolerance (67.5%, 27/40) ahd accumulated visceral fat, high insulin resistance, and low BMI (6). Metformin, thiazolidine, dipeptidyl peptidase-4 inhibitor, or glucagon-like peptide-1 receptor agonists could be carried out for diabetes treatment in Werner syndrome (7). Insulin, metformin tablets, and pioglitazone dispersible tablets were used in our patient, and for 18 months of treatment, there were no adverse reactions. The probability of skin ulcers in Werner syndrome was about 40%, and ulcers were often located at the distal one-third of the lower legs (8). Skin ulcers in Werner syndrome patients might be a double effect of the *WRN* gene abnormality and poor blood glucose control. Moreover, this may partly be because osteogenesis-related gene expression is upregulated while adipogenic and chondrogenic genes are downregulated in dermal fibroblasts from the foot in comparison with the trunk, resulting in uneven distribution of fat and skin ulcers in Werner syndrome (9). Nonsurgery, surgery, or invasive surgery should be considered for ulcer treatments under appropriate conditions, and anti-infection should be given more attention. The dressing of functional peptide (SR-0379) was proved safe, well-tolerated, and effective for leg ulcers in Werner syndrome, and the reduction rate of ulcer size was 22.9% in men after 4 weeks of treatment (*n* = 4) (10). In our patient, the ulcers in the foot were treated with skin flap repair, and some recovery was obtained after 40 days of dressing change cure.

MDS is a kind of clonal disorder in hematopoietic stem cell progenitors, with ineffective hematopoiesis, morphologic dysplasia, and a high risk of acute myeloid leukemia (AML). Almost 90% of MDS patients were detected carrying a somatic mutation in at least one gene, and the mutation genes were mainly implicated in the pathways of RNA splicing, DNA damage response, epigenetic regulators, signal transduction, and transcription factors (11). Werner syndrome patients with *WRN* mutations were easy to obtain *p53* gene mutations or related chromosomal abnormalities, and this was different from other MDS/AML patients’ changes in cellular or molecular genetic material. In hematopoietic stem cells, WRN function loss could result in p53 inactivation and acquirer of competitive fitness and then develop into myeloid malignancies (12), which may increase chemosensitivity. CHK1-related homologous recombination repair (HRR) in WRN defection cells was the key approach for repairing double-strand breaks (DSB) caused by ionizing radiation, which resulted in hyper-radiosensitization (13). The specific molecular mechanism of tumorigenesis in Werner syndrome patients may lead to therapeutic differences.

The treatments for MDS in Werner syndrome were very limited. Chemotherapy could be difficult for Werner syndrome patients, and a 44-year-old male Werner syndrome patient with AML was treated using a combined chemotherapy regimen of cytarabine, mitoxantrone, and etoposide, while the abnormal toxicity occurred on day 5, which did not occur in other patients with the same treatment regimen (14). Cord blood transplantation (CBT) has some effects on the MDS of Werner syndrome. A 44-year-old male patient with MDS underwent CBT and obtained a 15-month survival period, during which time he had MDS remission and no treatment-related toxicity (15). Allogeneic hematopoietic cell transplantation (HCT) may be feasible for Werner syndrome-related AML, and an 18-year-old female patient with AML obtained a 5-year survival after HCT without severe chemotherapy or transplant-induced toxicities (16). Anemia is one of the major concerns in MDS patients. Transfusions and erythropoiesis-stimulating agents could be used. MDS accounted for 7.2% of all transfused patients, which was the second cause of hematologic prescriptions in Europe and the USA (17). In this study, the patient just received supportive treatment such as blood transfusion, and the effect was limited. In general, the *WRN* gene mutation made the treatment of MDS in Werner syndrome very difficult, and the effects of supportive or chemotherapy were very few, while CBT and HCT may have better effects.

In conclusion, we report a Chinese Werner syndrome male patient with diabetic foot ulcers and MDS. The c.3744dupA mutation of the *WRN* gene in our patient was a novel pathogenic variation for Werner syndrome.

## Data availability statement

The original contributions presented in the study are included in the article/supplementary material. Further inquiries can be directed to the corresponding authors.

## Ethics Statement

The studies involving human participants were reviewed and approved by the ethics committee of the First Affiliated Hospital of Henan University of Science and Technology. The patients/participants provided their written informed consent to participate in this study. Written informed consent was obtained from the individual(s) for the publication of any potentially identifiable images or data included in this article.

## Author contributions

HP, HJ, and HZ contributed to the conception and design of the study. HP and JW wrote the draft of the manuscript. YL, HY, LL, and YM collect data. All authors contributed to manuscript revision and read and approved the submitted version.

## Funding

This work was supported by grants from the Joint Co-construction Project of Henan Medical Science and Technology Research Plan (No. LHGJ20210598) and The Science and Technology Plan Medical and Health Project in Luoyang (No. 2101048A).

## Conflict of Interest

The authors declare that the research was conducted in the absence of any commercial or financial relationships that could be construed as a potential conflict of interest.

## Publisher’s note

All claims expressed in this article are solely those of the authors and do not necessarily represent those of their affiliated organizations, or those of the publisher, the editors and the reviewers. Any product that may be evaluated in this article, or claim that may be made by its manufacturer, is not guaranteed or endorsed by the publisher.
